# Observations at the Bedside in a Number of Cases and Diseases

**Published:** 1887-01-01

**Authors:** S. B. Bouchelle

**Affiliations:** Thomasville, Ga.


### OBSERVATIONS AT THE BEDSIDE IN A NUMBER OF CASES AND DISEASES.

By S. B. BOUCHELLE, M. D., OF THOMASVILLE, GA.

*Obstetrics.*— In 1876 I was called to Mrs. R., who had been suffering for seven hours with intermittent pains in the head. No pain in any other region. It was about time for delivery according to her count, hence it was deemed best to ascertain the condition of the os uteri. It was found dilated, and dilating, as these periodic pains recurred in the head. Here was an anomaly ; no pain in the back and none in the region of the uterus, but excruciating pains in the head, and the uterus responding. What was to be done ? With no history of such a case in the books or journals my perplexity can well be imagined. After a few moments of confused deliberation, grs. 25 of chloral were administered, and in twenty minutes all pains in the head ceased, and then they were in back and uterus, and so continued till delivery was effected. Why was it thus ?

Case 2.— In 1884 was called to Mrs. M., also enciente; found her suffering with periodic pains in the right side of head and face simulating neuralgia. She had contracted a cold and had bad teeth on that side, so neuralgia was diagnosed. A full dose of morphia was administered after examining the os uteri and finding it firmly closed. It was nearing her time for confinement. Left her at 8 o'clock a. m. entirely relieved of all pain. At 11 o'clock I was sent for, and being absent, my friend Dr. B. was called in, and found her breathing heavily after a convulsion. He examined the os and found it still closed. What I had done was reported to him, and he diagnosed morphine poison, and administered quinine and coffee and sent for another friend, Dr. T. By this time another convulsion came on. Dr. T. examined and found the mouth of the womb rapidly yielding, and entered a counter diagnosis of puerperal convulsions. They together did all they could to expedite labor, and in four hours delivered her of a fine boy. But the convulsions still came. Morphine and chloral was administered freely and repeatedly till about 6 o'clock p. m. Dr. B. called on me as soon as I returned, and reported the details as above given, and a messenger came for me to see her again. I found her in a semi-comatose condition, and breathing heavily, and considerable jactitation. I repeated chloral by enema with unsatisfactory results. Then chloroform was given freely, with evanescent effects. At 1 o'clock convulsions supervened very violently. Then three drops of croton oil were administered, which operated in twenty minutes. Then there was a cessation of every outward symptom. There was no more tossing, and convulsions returned no more. Would the croton oil have had this effect if it had been administered prior to all other remedies? Let it be remarked that there was no blood letting in this case, and, further, that croton oil has had the same gratifying result in two or three other cases where everything else failed, even bleeding.

*Quinine in Enciente Patients.*— I have, in at least a score of cases, in that many years, administered quinine in all stages of utero-gestation with not a single untoward result. How do you manage at the culminating point of delivery? Support the peræneum with the right hand, till the head is born, then with both hands take hold of the head and make gentle traction, and in two seconds the body is delivered. After attention to the cord — see that is not about the child's neck — then immerse the left hand freely in cold water and apply over the region of the womb, and repeat till I can feel contraction in response. I then tie the cord twice, cut it between the ligatures, deliver the little squealer to a competent nurse, with directions to grease it all over well with lard and wash well with mild soap. Then I apply the left hand over the womb externally, and introduce the right hand and deliver the placenta. Again, I immerse the left hand in cold water and apply and manipulate till I feel the womb well knotted up in the illiac fossa. *All this is done in less than ten minutes.* I never let a nurse attend to the umbilical cord, but have a soft cloth — can't always get linen — fold once, cut a hole, saturate it well with lard, pass the cord end through hole, wrap and fold it in the cloth, turn to the right across the abdomen and apply the bandage, and see it pinned well and tight. Then I am done with the child. What more for the mother? See all soiled garments and bed clothes removed, apply a bandage and give some nutrient drink, and bid her rest as quietly as she can till the following morning. Then I invariably administer a full dose of oil and turpentine. I call two tablespoonfuls of oil and one of turpentine a full dose. After this operates, I dismiss the case. In an active practice of nearly thirty years I have pursued this regime, and never lost but one case of a lying-in woman, and she had taken a big dose of salts before she sent for me. She had a safe delivery, but diarrhœa set in and nothing we could do had any effect. She had had no trouble in that direction before the salts were taken. How much did the salts have to do with this result?

*What About Forceps?* — I think well of them, if left out of the reach of the accoucheur, in about four hundred and ninety-nine cases out of every five hundred. I have had a great variety of obliquities in presentation, but never have resorted to them. I name these cases — the long diameter of the head to the short diameter of the pelvis — several times, relieved them by manual manipulation. Abdomen, back, side and shoulder; also arm and one foot, the other lodged above the symphysis pubic. Relieved by manipulating and turning.

*What About Chloroform?* — I never give it. What do you do in cases of inertia uteri? Very seldom give ergot; often give chloral. Does it superinduce uterine contraction? Yes. What is the rationale? I don't know. What preparation of ergot do you use? I prefer the wine to any of the preparations. Why? Because it doese not deteriorate by keeping, and does not offend the stomach of the sufferer. If you do not give it to invite uterine contractions often, when do you give it? Invariably just after the delivery of the placenta. Why? To guard against post partum hemorrhage. Are your patients ever troubled with this complication? No, if I leave no part of the placenta or membranes, and this, of late years, I never do. When in the crysalis stage of an accoucheur, this sometimes occurred because of timidity. *Suaviter in modo, et fortiter in re*, is here more needed than almost anywhere in a physician's life. Does the placenta ever cohere unnaturally to the walls of the uterus? Yes, often. Do you wait for spontaneous separation? No; but introduce the hand into the uterus, the left hand applied over the outer wall, and, with the fingers, gently but persistenly pry it loose, and then bring it away as the hand is withdrawn. Let it be noted that immediate delivery of the placenta and decidua removes all danger of hour-glass contractions, and also post partum hemorrhage. When may we expect puerperal fevers? Only when the subject has been suffering with intermittent or remittent fever, anterior to accouchment. What do you do for puerperal fever? Poultice freely; give opium, blue mass, and apply spirits of turpentine freely to back and bowels, and give 10 to 20 drops three times a day in a little water or sweet milk.

